# Exploring the Post Mortem Interval (PMI) Estimation Model by circRNA *circRnf169* in Mouse Liver Tissue

**DOI:** 10.3390/ijms26031046

**Published:** 2025-01-26

**Authors:** Jiewen Fu, Binghui Song, Jie Qian, Jingliang Cheng, Sawitree Chiampanichayakul, Songyot Anuchapreeda, Junjiang Fu

**Affiliations:** 1 Key Laboratory of Epigenetics and Oncology, The Research Center for Preclinical Medicine, Southwest Medical University, Luzhou 646000, China; fujiewen@swmu.edu.cn (J.F.); songbinghui@stu.swmu.edu.cn (B.S.); 20210199120039@stu.swmu.edu.cn (J.Q.); jingliangc@swmu.edu.cn (J.C.); 2 Laboratory of Precision Medicine and DNA Forensic Medicine, The Research Center for Preclinical Medicine, Southwest Medical University, Luzhou 646000, China; 3 Department of Medical Technology, Faculty of Associated Medical Sciences, Chiang Mai University, Chiang Mai 50200, Thailand; sawitree.chiampa@cmu.ac.th; 4 Center of Excellence in Pharmaceutical Nanotechnology, Chiang Mai University, Chiang Mai 50200, Thailand; 5 Laboratory of Forensic DNA, The Judicial Authentication Center, Southwest Medical University, Luzhou 646000, China

**Keywords:** post mortem interval (PMI), circular RNA (circRNA), forensic biomarker, post mortem interval estimation, forensic pathology

## Abstract

Estimating the post mortem interval (PMI) is a crucial and contentious issue in forensic research, particularly in criminal cases. Traditional methods for PMI estimation are limited by constraints and inaccuracies. Circular RNA (circRNA), formed through exon or intron looping to create a complete circular structure without a 5′ end cap and a 3′ poly(A) tail, exhibits exceptional stability, abundance, and tissue-specific characteristics that make it potentially valuable for PMI estimation. However, research on the exploration or application of circRNA in PMI estimation has been limited. This study aims to investigate the correlation between circRNA and PMI. In this study, liver tissue samples were collected from mice at six different time points at 4 °C, 18 °C, 25 °C, and 35 °C, respectively. The reference gene *28S* rRNA and the biomarker *circRnf169* were successfully screened. Quantitative PCR was employed to examine the correlation between *circRnf169* levels and PMI. At 4 °C, the level of *circRnf169* decreased with prolonged PMI, whereas at 18 °C, 25 °C, and 35 °C, the *circRnf169* RNA was degraded rapidly, indicating that *circRnf169* is suitable for PMI estimation at low temperatures or early PMI. These findings suggest the establishment of mathematical model for early PMI based on *circRnf169* using liver tissue, which may serve as a reliable marker. Further research is required in order to develop more markers in mice and/or to validate these mathematical models in human samples.

## 1. Introduction

The post mortem interval (PMI) refers to the time interval spanning from the occurrence of death to the examination of the corpse upon discovery. The accurate determination of the time of death holds paramount significance in forensic identification, particularly in the investigation of homicide cases, as the time of death is often closely associated with the occurrence time of the case. Among them, traditional PMI determination is classified into early death determination and late death determination. The methods of early death determination encompass body temperature measurement, livor mortis, rigor mortis, corneal alterations, the extent of digestion of stomach and intestinal contents, and bladder urine volume, etc. The methods of late death determination include the degree of body decomposition, data on necrophagous insects, and decomposed to bony skeleton corpses, etc. However, early PMI estimation methods based on traditional approaches are often constrained and inaccurate.

With the advancement of science and technology, the methods for PMI estimation have become increasingly precise and diversified, offering new horizons and approaches for the determination of PMI. PMI can be estimated by the changes in the microbial community on the cadaver after death, and then artificial intelligence is used to estimate its evolution rule [1,2]. Raman spectroscopy is applied to estimating the PMI of human skeletal remains [3], or DNA and RNA are extracted from the cadaver to observe their degradation rule, so as to infer the PMI of the cadaver [4,5]. DNA is more stable and not easily degraded than RNA, which is more suitable for long-term PMI estimation, while RNA is more suitable for short-term PMI estimation because of its easy degradation. Moreover, at present, there are already a large number of research articles related to the use of RNA (such as reference genes) for PMI estimation [6,7,8].

RNA can be roughly divided into linear RNA and circular RNA (circRNA). Compared with linear RNA, circRNA does not have a 5′ terminal cap and 3′ terminal poly (A) tail and joins the 3′ and 5′ ends to form an entire circular structure through exon cyclization or intron cyclization [9,10,11]. CircRNA avoids degradation by nucleases and is therefore less susceptible to RNase degradation than linear RNA. In addition, circRNA has the characteristics of high stability, high abundance, and tissue specificity [12,13], making circRNA potentially valuable in PMI estimation. However, there are few studies on circRNA in PMI estimation at present [14].

The liver, recognized as the largest solid organ in the human body, plays an indispensable role in a myriad of physiological processes, including metabolism, synthesis, digestion, detoxification, and hematopoiesis [15,16,17]. Recent investigations into circRNAs within hepatic tissue have yielded significant insights. Liu et al. [18] demonstrated that *circASH2* disrupts the cytoskeletal structure of tumor cells and diminishes cell adhesion, thereby inhibiting metastasis in liver cancer. Zhao et al. [19] reported that *circRNA-007371* promotes angiogenesis by a miRNA sponge mechanism during liver fibrosis. Guo et al. [20] identified that *circRNA-14723* promotes hepatocyte proliferation during rat liver regeneration through its activity as a sponge for *rno-miR-16-5p*. Despite extensive research on various facets of circRNAs in the liver, their potential application for PMI estimation has received scant attention.

In this study, we aimed to enhance our understanding of circRNAs in hepatic research by investigating the relationship between the biomarker *mmu-Rnf169_0002* (*circRnf169*) and PMI estimation in liver tissue samples. Mouse liver tissues were collected at different time intervals under controlled conditions at 4 °C (low temperature), 18 °C (room temperature), 25 °C (ambient temperature), and 35 °C (high temperature). Subsequently, the candidate biomarkers were screened and selected using an online platform, followed by semi-quantitative polymerase chain reaction (PCR) to evaluate *circRnf169* levels post mortem. The mathematical model for PMI estimation utilizing *circRnf169* at 4 °C was established and further validated against known deceased samples to confirm its reliability.

## 2. Results

### 2.1. Screening of Reference Genes

**Figure 1:**
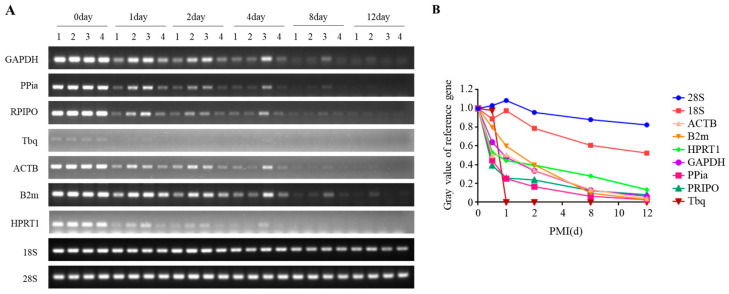
The levels of the reference gene at day 0, 1, 2, 4, 8, and 12 in liver tissue at 4 °C. (**A**) The levels of reference genes in semi-quantitative PCR experiments at different PMIs. (**B**) The quantitative results of panel (**A**). d, days.

In this study, primers were designed using Primer 3.0 (Table 1), and we improved their specificity using the NCBI primer-blast tool. We initially selected the most suitable internal reference gene by screening nine different genes in the internal control genes (ICG) database. Based on the mouse liver tissues at different PMIs, which were maintained at 4 °C, we observed that, except for *18S* ribosomal RNA (rRNA) and *28S* rRNA, all other messenger RNAs (mRNAs) degraded progressively over time (Figure 1A). Especially on the 12th day, mRNAs were hardly visible. Then, we made a line chart (Figure 1B) to visualize the stability of each gene more intuitively. Only *18S* rRNA and *28S* rRNA were relatively stable, especially *28S* rRNA, which was the most stable marker. Therefore, we selected *28S* rRNA as the internal reference gene.

### 2.2. Selection and Validation of the Biomarker in Liver Tissue

**Figure 2:**
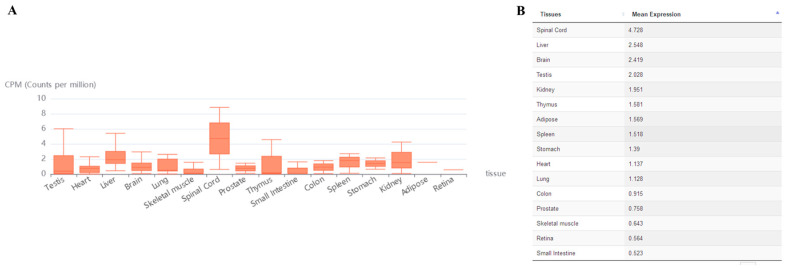
Expression of circRnf169 in mouse multiple tissues. (**A**) Expression of circRnf169 in 16 different tissues. (**B**) The expression quantity of panel (**A**) is shown in detail.

**Figure 3:**
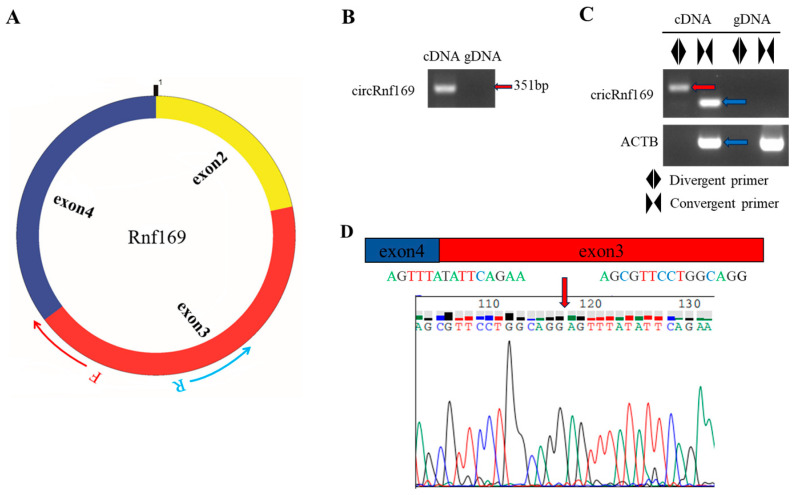
Validation of circRnf169 in the mouse liver tissue. (**A**) Schematic diagram of the structure of circRnf169 and the direction of primer amplification. (**B**) PCR amplification of circRnf169 in cDNA and gDNA using divergent primers. (**C**) Amplification results of convergent primers and divergent primers of circRnf169 and the reference gene *ACTB* in cDNA and gDNA. The red arrow indicates the band produced by the divergent primer, while the blue arrow denotes the band generated by the convergent primer. (**D**) Sanger sequencing results of circRnf169. Different colored fonts and lines represent different bases. This subfigure only shows the most critical part of Sanger sequencing, with the red arrow indicating the splice site of circRnf169.

The circAtlas 3.0 online website was selected to select the high abundance and conserved circular RNA i.e., *cricRnf169*. The abundances of the *cricRnf169* in each tissue are shown in Figure 2A,B. CircPrimer 2.0 software was used to determine the location of the *circRnf169* primer (Figure 3A). The amplification results of *circRnf169* divergent primers can only be seen in the cDNA sample (Figure 3B). In addition, convergent primers were also used to amplify both cDNA and genomic DNA (gDNA) samples. The results indicated that the convergent primers of *ACTB* had bands (blue arrow) in cDNA and gDNA samples, while the convergent primers of *circRnf169* only had a band in the cDNA sample. However, among the divergent primers, only the *circRnf169* had the band (red arrow) in the cDNA, which verified the specificity of the circular primers and the circular structure (Figure 3C). Since circular RNA is an end-to-end sequence, it can be amplified by both convergent and divergent primers in the cDNA samples. However, no bands can be amplified by divergent primers in the gDNA samples, indicating the high specificity of the primers. Moreover, only the convergent primer of *ACTB* can be amplified in both cDNA and gDNA samples, because it is a linear structure, while the divergent primers cannot amplify any bands. At the same time, to verify the back-splice junction of *circRnf169*, Sanger sequencing was performed for confirmation. The results showed that it was consistent with the database sequence and shared the same splice site (red arrow).

### 2.3. Construction of the Mouse PMI Estimation Models by Semi-Quantitative PCR

**Figure 4:**
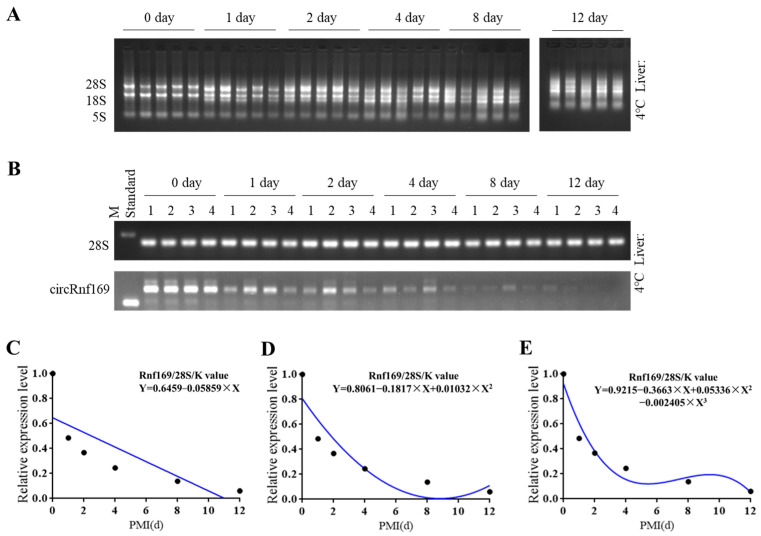
Construction of PMI estimation models based on mouse liver tissues at 4 °C. (**A**) Quality of total RNA at different PMIs including 0 d, 1 d, 2 d, 4 d, 8 d, and 12 d. (**B**) Results of semi-quantitative PCR assessing the expression levels of circRnf169 and *28S* rRNA in liver tissues at 4 °C on day 0, 1, 2, 4, 8, and 12. (**C**–**E**) The linear equation, quadratic equation, and cubic equation were built at 4 °C. d, days.

Samples were extracted at 4 °C across 6 PMIs (day 0, 1, 2, 4, 8, and 12), with 5 mouse liver tissue samples in each group. The quality and integrity of total RNA were shown in the results of the agarose gel (Figure 4A). The results showed that the RNA degradation rate in the liver occurred relatively rapidly, beginning on the first day and progressing over time. Even under low-temperature conditions, the degradation of total RNA was still rapid due to the abundance of RNA-degrading enzymes in the liver. To construct a PMI estimation model at 4 °C, *28S* rRNA was used as the internal reference gene, and *circRnf169* was selected as the biomarker. One sample showing a significant difference was excluded from each group of 4 °C samples, leaving 4 samples were subjected to semi-quantitative PCR (Figure 4B). Figure 4 shows that the overall *circRnf169* degraded continuously over time, with significant degradation occurring between day 0 and day 1, followed by slow degradation. The equation was constructed based on the ratio of the gray value of the biomarker to the gray value of the reference gene at different PMIs (Figure 4C–E).

*28S* rRNA was selected as the internal reference gene of semi-quantitative PCR analysis and constructed three equations, including a first-order equation, a quadratic equation, and a cubic equation. The K value was calculated from the ratio of the 0-day cricRNA gray value to the corresponding 0-day reference gene gray value to be 0.2802. The R^2^ and *p* values of the three equations were 0.6423 (*p* = 0.055), 0.8395 (*p* = 0.064), and 0.9468 (*p* = 0.079), respectively. The R^2^ values of the three equations were greater than 0.6, but only the *p* value of the linear equation was less than 0.05 (Table 2).

**Figure 5:**
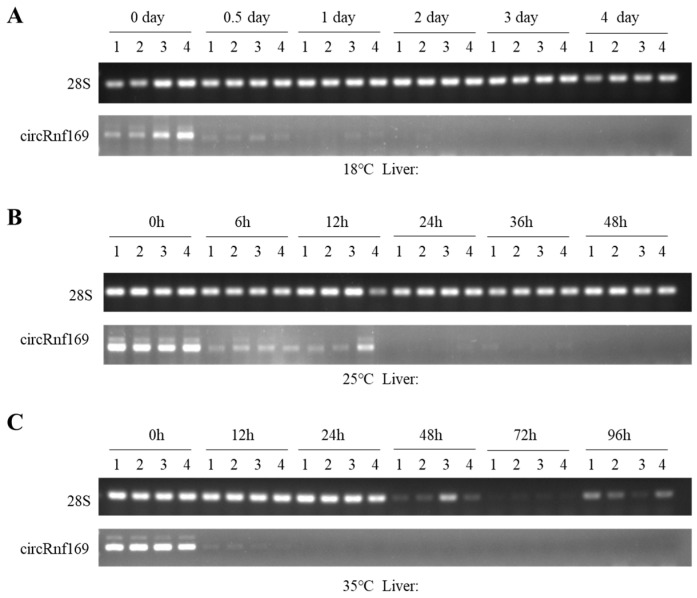
Semi-quantitative PCR results of circRnf169 and *28S* rRNA levels at different temperatures. (**A**) Levels of circRnf169 and *28S* rRNA at 18 °C on day 0, 0.5, 1, 2, 3, and 4. (**B**) Levels of circRnf169 and *28S* rRNA at 25 °C at 0 h, 6 h, 12 h, 24 h, 36 h, and 48 h. (**C**) Levels of circRnf169 and *28S* rRNA at 35 °C at 0 h, 12 h, 24 h, 24 h, 48 h, 72 h, and 96 h. h, hours.

At 18 °C, RNA was extracted from liver tissues at 6 time periods at day 0, 0.5, 1, 2, 3 and 4 after death. At 25 °C, RNA was extracted from liver tissues at 0 h, 6 h, 12 h, 24 h, 36 h, and 48 h postmortem. At 35 °C, RNA was extracted from liver tissues at 0 h, 12 h, 24 h, 36 h, 48 h, and 96 h postmortem. The results of agarose electrophoresis showed that *circRnf169* degraded rapidly after 0 h at the three temperatures and the degradation rate continued to accelerate with the increase in temperature (Figure 5A–C). Because *circRnf169* degraded too quickly at room temperature and at a high temperature to construct equations, we only present its agarose electrophoresis results.

### 2.4. Validation of the PMI Estimation Model

**Figure 6:**
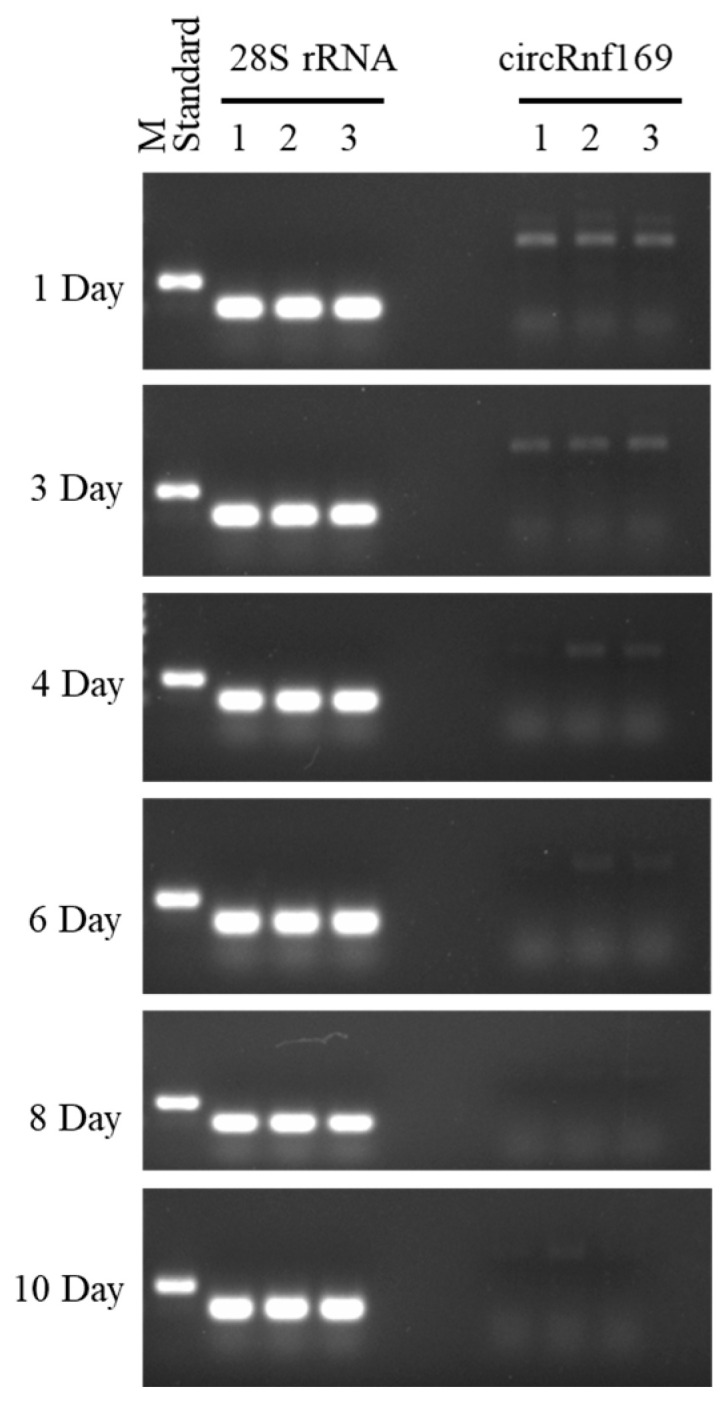
The validation of the mouse PMI estimation model based on the samples of liver tissues at 4 °C at different PMIs, including 1 d, 3 d, 4 d, 6 d, 8 d, and 10 d.

To further verify the accuracy of the PMI estimation model, a double-blind method was applied. Samples with known PMI were used, consisting of three groups (1 d, 4 d, and 8 d) that matched the same time points used to establish the equation and three other groups (3 d, 6 d, and 10 d) that did not match. The results of semi-quantitative PCR experiments to validate the mathematical model are shown in Figure 6. Based on the error rates, the accuracy of the three equations was evaluated (Table 3). The results showed that the error rates for the validation samples at the matched time of the equation were 200%, 110%, and 60% for the 1-day sample; 150%, 65%, and 22.5% for the 4-day sample; and 25%, 18.8%, and 11.3% for the 8-day sample. In addition, the error rates for the validation samples at the non-matched time of the equation were 140%, 40%, and 3.3% for the 3-day sample; 75%, 21.7%, and 21.7% for the 6-day sample; and 2%, 8%, and 21% for the 10-day sample. These results indicated that the cubic equation had the smallest error and was closest to the real PMI, and may be more suitable for PMI estimation at 4 °C.

## 3. Discussion

The estimation of PMI can be categorized into early and late PMI estimation. Early PMI estimation relies on post mortem phenomena such as rigor mortis, livor mortis, and body temperature [21], whereas late PMI estimation is based on the extent of decomposition, forensic entomology, and forensic anthropology [22,23]. However, conventional methods for estimating PMI are often limited in accuracy and more susceptible to temperature variations. Accurate PMI assessment is crucial for forensic identification and criminal investigations. Currently, molecular techniques are employed as new tools for this purpose. Both DNA and RNA have been explored as new biomarkers in PMI estimation. DNA is more suitable for late PMI estimation due to its stability, while RNA is usually used for early PMI estimation because it is easily degraded by RNases [24,25]. For PMI estimation, an ideal forensic biomarker should exhibit specificity, stability, and high sensitivity [26]. CircRNA has a unique circular structure and demonstrates greater stability compared to other types of RNA.

CircRNA has emerged as a prominent area of research due to its potential applications as a therapeutic target, prognostic marker of cancer treatment, and diagnostic indicator of liver diseases. Presently, circRNA is studied in forensic medicine. It can facilitate age estimation of human specimens lacking morphological features at crime scenes while serving as a promising biomarker for forensic age prediction [27]. Furthermore, circRNA may help in identifying various bodily fluids through tissue specificity [28]. Multiple circRNAs were screened in the pre-experiment in the mouse liver. Subsequently, we found that, compared with cricRnf169, these circRNAs, for example *circAass*, exhibited lower expression levels and reduced stability in the liver. When evaluated for combined applications, their performance was comparable to that of a single circular RNA (*cricRnf169*), without showing more improvements. Moreover, equations constructed for combined applications revealed that their R^2^ values were lower than those of cricRnf169, indicating that they did not exhibit better goodness of fit and *p*-values compared with *cricRnf169*. More studies involving the selection of more cirRNA should be conducted in the near future. Nevertheless, in this study, a potential biomarker, cricRnf169, was found for PMI estimation.

The *Rnf169* gene has been implicated in DNA repair mechanisms and plays a role in negatively regulating cellular responses to DNA damage along with double-strand break repair processes [29]. Wang et al. found that non-coding RNA (ncRNA)-mediated upregulation of *Rnf169* expression could influence immune cell infiltration within the microenvironment. Notably, high levels of *Rnf169* expression correlate with poor prognosis in pancreatic cancer cases [30].

Stable internal reference genes are needed when exploring methods to accurately estimate PMI. Thus, we selected nine candidate internal reference genes frequently used from the ICG database [31,32] and employed semi-quantitative PCR experiments to assess their stability. The validation of the PMI estimation model showed that the error rates were lower when using linear equations for longer PMIs, while the error rates were always the lowest when the cubic equation was used, except for the 10th day. This indicates that all equation models can be further optimized.

In this study, the potential of *circRnf169* in estimating the early and late PMIs in the liver tissue at low temperature is demonstrated, and it also possesses the potential for estimating the early PMIs at room temperature and high temperature. This effectively supplements the research on PMI estimation at low temperatures. Furthermore, this study is merely an exploration of circular RNA in mouse PMI estimation. Since no human samples were involved, it is difficult to directly apply them to PMI estimation in human forensic medicine. The subsequent experiments will require the participation of human samples to validate the model. Due to the abundance of enzymes in the liver, *circRnf169* was degraded by approximately half within just one day at a low temperature of 4 °C. This was one of the reasons for its rapid degradation at room temperature and high temperature.

In addition, more circRNAs can be screened as biomarkers for PMI estimation, which will contribute to improving the accuracy of the model at aforementioned. The combined application of multiple circRNAs from a single tissue or the combined application of circRNAs from multiple tissues will be the future development direction of PMI estimation in forensic medicine. For instance, the brain is in a closed condition and is less influenced by the external environment. Thus, PMI estimation can be achieved over a long period of time and within a certain time at the same temperature. Furthermore, the selection of appropriate tissues at different temperatures can better establish the model equations. For example, the rapid degradation of *circRnf169* at room and high temperatures makes it more suitable for estimating early PMIs, enabling more precise time estimates.

## 4. Materials and Methods

### 4.1. Sample Collection

Nine-week-old male BALB/c mice (23–26 g) were used for this study. All animal experiments were performed in accordance with international, national, and institutional animal care guidelines. This study was reviewed and authorized by the Animal Ethics Committee of Southwest Medical University, Sichuan, China (approval number: 20230821-011). The mouse was purchased from Tengxin Biotechnology Co., Ltd. (Chongqing, China). The mice were kept at a temperature range of 20–26 °C and humidity between 40 and 70%. After anesthesia, the mice were euthanized, and 5 liver tissue samples were collected at different time points post mortem. There were 4 different temperatures, including 4 °C, 18 °C, 25 °C, and 35 °C, and each temperature had 6 different time points. The details are as follows: 4 °C (day 0, 1, 2, 4, 8, and 12), 18 °C (day 0, 0.5, 1, 2, 3, 4), 25 °C (0 h, 6 h, 12 h, 24 h, 36 h, 48 h), and 35 °C (0 h, 12 h, 24 h, 48 h, 72 h, 96 h).

### 4.2. Total RNA Extraction

A total RNA extraction kit (Tiangen Biochemical Technology Co., Ltd., Beijing, China) was used to extract total RNA from mouse liver tissues. According to the manufacturer’s instructions, the total RNA was successfully extracted. The RNA concentration was then measured using a spectrophotometer (ND-2000, Nanodrop Technologies, Wilmington, DE, USA), and the RNA quality was detected by agarose electrophoresis.

### 4.3. Reverse Transcription

The ReverTra Ace qPCR RT Master Mix (Oriental Textile Biotechnology Co., Ltd., Osaka, Japan) was used to reverse transcript RNA into cDNA. The reverse transcription system was as follows: 5× RT Master Mix 2 μL, RNA 1 μg, and nuclease-free water added to 10 μL. The PCR program was as follows: 37 °C 15 min, 50 °C 5 min, 98 °C 5 min, 16 °C maintenance. When the reverse transcription finished, the cDNA was diluted with water. Afterwards, it was stored at −20 °C for further experiments.

### 4.4. Selection of Circular RNAs and Internal Reference Genes

The online website circAtlas 3.0 (https://ngdc.cncb.ac.cn/circatlas/index.php (accessed on 15 December 2023)) was used to find the candidate circRNAs in the mice. In circAtlas 3.0, the top 30 circRNAs with high expression levels in the mouse livers were selected. CircRNAs lacking sequence information or with excessively short sequences were excluded. Appropriate circRNAs, including *circRnf169* from multiple circRNAs such as *circARCN1_003*, *circAPOB_012*, *circARIH1_010*, *mmu-Rnf169_0002*, *mmu-Asph_0007*, *mmu-Aass_0001*, *circFBXW4*, etc., were screened out, followed by experimental verification. For the selection of internal reference genes, we used the ICG (2.0) website (https://ngdc.cncb.ac.cn/icg/ (accessed on 14 October 2023)) to find some internal reference genes with highly frequent usage. The internal reference genes needed in this experiment were screened by measuring their expression changes at different times of death.

### 4.5. Primer Design and Semi-Quantitative PCR

The primer 3.0 (https://primer3.ut.ee/ (accessed on 15 December 2023)) was applied for online website primer design. Divergent primers and convergent primers were designed for the circRNA. Divergent primers can be used to amplify circular RNA, and convergent primers can be used to amplify linear RNA. The details of the PCR amplification program were as follows: Pre-denaturation at 95 °C for 90 s, denaturation at 95 °C for 30 s, annealing at 63 °C for 30 s, extension at 72 °C for 20 s, a total of 30 cycles for circRNA and 20 cycles for the internal reference gene, and final extension at 72 °C for 5 min. The amplification product was maintained at 16 °C. Then, the agarose electrophoresis was performed. In addition, to maintain the consistency of the exposure conditions for agarose electrophoresis, 2 μL of the standard DNA with known concentration was used in each electrophoresis to reduce the influence of other factors. The standard DNA was obtained by PCR amplification of mouse liver tissue gDNA at a concentration of 600 ng using *GAPDH* primer pairs.

### 4.6. Data Analysis

Photoshop software with version 2022 V23.4.1 (Adobe Systems Inc., San Jose, CA, USA) was used to analyze the background and band gray values on agarose gel and subtracted the background gray value from the band gray value to remove the background influence. Then, we calibrated the exposure condition differences that existed between the standard products. The gray values of the biomarker and the reference gene were standardized after normalization. The average gray value of the biomarker at each time point was divided by the average gray value of the reference gene band to determine the relative level of the PCR product for each group. The ratio of the 0-day cricRNA gray value to the corresponding 0-day reference gene gray value was defined as the K value. We constructed an equation as follows: Y = (cricRNA n-d gray value − cricRNA n-d background gray value)/(reference gene n-d gray value − reference gene n-d background gray value)/K.

## 5. Conclusions

This study found that *circRnf169* in liver tissue degraded continuously over time at 4 °C. A mathematical model was successfully established based on the biomarker at low temperatures of 4 °C. Since *circRnf169* degrades quickly at room and high temperatures, it may be suitable for early PMI estimation. However, its mathematical model still requires further verification using human tissue samples.

## Figures and Tables

**Table 1:** The primers of *28S* rRNA, ACTB, and circRnf169.

Marker	Size (bp)	Forward Primer (5′-3′)	Reverse Primer (5′-3′)
circRnf169-divergent	351	CTGAAAACAAGTCTGGAGCAG	TCAGCTTTCCTCTTCCCCATT
cricRnf169-convergent	200	ATCAAGTTAAGCAAGCCGGG	TGCTCCAGACTTGTTTTCAGC
ACTB-divergent	190	CTGGCCTGTACACTGACTTGA	GTCATCCATGGCGAACTGGT
ACTB-convergent	392	TGTTACCAACTGGGACGACA	TCTCAGCTGTGGTGGTGAAG
*28S* rRNA	105	TCATCAGACCCCAGAAAAGG	GTTGATTCGGCAGGTGAGTT

**Table 2:** Mathematical models of PMI estimation.

Temperature	Reference Gene	Mathematical Models	R^2^	*p*-Value	Equation
4 °C	*28S* rRNA	Y = 0.6459 − 0.05859 × X	0.6423	0.055	Linear
		Y = 0.8061 − 0.1817 × X + 0.01032 × X^2^	0.8395	0.064	Quadratic
		Y = 0.9215 − 0.3663 × X + 0.05336 × X^2^ − 0.002405 × X^3^	0.9468	0.079	Cubic

**Table 3:** Validation of the mathematical models by semi-quantitative PCR.

Temperature	Reference Gene	Real PMI	Estimation PMI	Estimation Error	Error Rate (%)	Equation
4 °C	*28S* rRNA	1 d	3.0 d	2.0 d	200.0	Linear
		1 d	2.1 d	1.1 d	110.0	Quadratic
		1 d	1.6 d	0.6 d	60.0	Cubic
		3 d	7.2 d	4.2 d	140.0	Linear
		3 d	4.2 d	1.2 d	40.0	Quadratic
		3 d	3.1 d	0.1 d	3.3	Cubic
		4 d	10.0 d	6.0 d	150.0	Linear
		4 d	6.6 d	2.6 d	65.0	Quadratic
		4 d	3.1 d	0.9 d	22.5	Cubic
		6 d	10.5 d	4.5 d	75.0	Linear
		6 d	7.3 d	1.3 d	21.7	Quadratic
		6 d	7.3 d	1.3 d	21.7	Cubic
		8 d	10 d	2.0 d	25.0	Linear
		8 d	6.5 d	1.5 d	18.8	Quadratic
		8 d	7.1 d	0.9 d	11.3	Cubic
		10 d	10.2 d	0.2 d	2.0	Linear
		10 d	10.8 d	0.8 d	8.0	Quadratic
		10 d	12.1 d	2.1 d	21.0	Cubic

Note: d, days.

## Data Availability

All data used for the analyses in this report are available from the corresponding author on reasonable request.
